# Association and Distribution of Hypertension, Obesity and ABO Blood groups in Blood Donors

**Published:** 2012-09-22

**Authors:** Tulika Chandra, Ashish Gupta

**Affiliations:** 1 Department of Transfusion Medicine, King George’s Medical University, Lucknow, Uttar Pradesh, India.; 2 Department of Pathology, King George’s Medical University, Lucknow, Uttar Pradesh, India.

**Keywords:** Association, Hypertension, Obesity, Blood Donors

## Abstract

**Background:**

Hypertension is a major health problem, especially because it has no clear symptoms. It is strongly correlated with modifiable risk factors such as adiposities, age, stress, high salt intake. Overweight and obesity is conveniently determined from BMI and visceral adiposity is determined by waist circumference. ABO blood group is one such factor which needs to be investigated. The present study was performed to assess the association and distribution of hypertension, obesity, ABO blood groups in different categories of blood donors and its multipurpose future utilities for the health planners.

**Materials and Methods:**

A retrospective study was carried out on 23, 320 blood donors during a period of one year. All the blood donors were measured BMI, ABO blood group, systolic and diastolic blood pressure were determined and correlated for each other.

**Results:**

Hypertension of ABO blood group was B (8.7%) followed by group O (7.6%) group A (3.7%) and group AB (1.9%). In obesity of ABO blood group was B (7.9%) followed by group O (6.2%) group A (5.8%) and group AB (1.0%). Statistically significant difference was found in both groups (p < 0.001).

**Conclusion:**

The B blood group in blood donor was more susceptible to hypertension and obesity.

## Introduction

Hypertension is a major health problem, especially because it has no clear symptoms. Many people have hypertension without knowing it. It is now well proved that modifier factors like obesity, overweight that is measured by BMI, visceral adiposity measured by waist circumference, increasing age, are associated with the high prevalence of hypertension (1,5). The ABO blood group system was the first human blood group system discovered by Landsteiner in 1900. The ABO blood group system is the only system in which antibodies are consistently and predictably present in the serum of normal individuals whose red cells lack the antigens (6). The second type of blood group is the rhesus system. There are only two Rh phenotype such as Rh positive and Rh negative, depending on whether Rh antigen is present on the red cell or not. Determination of ABO blood groups is done by detecting A and B antigens. In addition, known red cells are used to detect anti-A and anti-B in the serum, by a process called ‘reverse’ grouping. ABO and Rh gene phenotypes vary widely across races and geographical boundaries (7,9) despite the fact that the antigens involved are stable throughout life. The resultant polymorphism remains important in population genetic studies, estimating the availability of compatible blood, evaluating the probability of hemolytic disease in the new born, resolving disputes in paternity/maternity and for forensic purposes (10, 11). The frequency of ABO and Rh phenotypes in different populations has been extensively studied. Different blood groups have been shown to be particularly associated with different diseases as well (12, 13). Rh system emerged as second most important blood group system due to hemolytic disease of newborn and its importance in Rh negative individuals in subsequent transfusions once they develop Rh antibodies (1). The present study was performed to assess the association and distribution of hypertension, obesity, ABO blood groups in different categories of blood donors and its multipurpose future utilities for the health planners.

## Materials and Methods

A retrospective study was carried out on 23, 320 blood donors (male and female) during a period of one year from 1^st^ January to 31^st^ December 2011 in the State Blood Bank, King George’s Medical University, Lucknow, India, for pediatric ward. The blood donors were selected after taking a detailed history and a complete examination regarding their eligibility criteria for blood donation. Donor’s name, age, sex, occupation, caste, complete postal address and contact number was taken. Donors were deferred or accepted according to their medical history regarding chronic or acute diseases. Findings were further confirmed by physical examination of the donor. Blood was taken from a donor only after fulfilling all the eligibility criteria of a healthy donor. Blood pressure was measured with digital Sphygmomanometer. Classification of hypertension was based on JNC guidelines (14) (a) Healthy blood pressure: < 120.80 (b) Pre-hypertension: between 120/80 and 140/90 (c) Hypertension: 140/90 or higher. BMI, which is the most commonly used indicator of obesity in population studies, was determined from calculated as weight in kilograms divided by height in meters squared (kg/m^2^) (15). Blood was taken for donors with hemoglobin more than 12.5 gm %. The donors have no history with any disease, infection or recent treatment. Written consent was also taken from them prior to donation regarding their acceptability for the tests to be carried out for the transfusion transmitted diseases. The Blood samples were obtained by standard procedures of venupuncture and subjected to determination of ABO and Rhesus blood group using antisera (Eryscreen Monoclonal ABO/Rh, Tulip Diagnostic Ltd. Goa, India) by combined slide and test tube methods. Each sample was tested for ABO and Rhesus status.

**Ethical Issue**

The donors signed an informed consent after being informed that the details of their blood groups will remain with blood bank and may be used either for research or transfusion purposes. This is a routine procedure and has been approved ethically by the drug licensing authorities of India. Documentation is an integral part of blood banking and the use of data for research purposes have been advocated, keeping the donors identity hidden. This study was carried out within the acceptable ethical norms.

**Statistical analysis**

Chi-square test was used. The confidence limit was kept at 95%, hence a P-value <0.05 was considered to be statistically significant.

## Results

The frequency of ABO and Rh blood groups in a total of 23,320 males and females, donor population was compared. Amongst Rh positive male donors blood group B was found to be most prevalent group (34.76%) followed by group O (29.57%), A (21.60%) and AB (14.06%). Amongst Rh positive female donors again blood group B was most common (35.29%) followed by group O (29.41%), A (20.58%) and AB (14.70%). Rh negative donors were 1060 (4.55%) amongst the total donors. On further analysis, female donors showed a relatively higher incidence of Rh negativity (10.53%) as compared to male (4.54%) (Table I). Among Rh negative male, blood group B (36.55%) was the commonest followed by group O (33.23%), A (19.41%) and AB (10.79%) whereas in Rh negative females, blood group B (50%) was followed by O and A (25% each). None of the female donors showed AB negative. The total of ABO blood group was group B (34.84%) followed by group O (29.75%) group A (21.50%) and group AB (13.91%). Distribution and prevalence of hypertension, obesity and overweight in blood donors were presented in Table II. The association between hypertension, obesity and ABO blood groups in blood donors is presented in Table III.

**Table I:** Comparison of Rh positive and Rh negative (%) between male and female donors

**Gender**	**No. of blood donors (%)**	**No. of Rh positive blood donors ** **(%)**	**No. of Rh negative blood donors ** **(%)**
**Male**	23282 (99.84%)	22226 (95.46%)	1056 (4.54%)
**Female**	38 (0.16%)	34 (89.47%)	4 (10.55%)
**Total**	23320	22260 (95.45%)	1060 (4.55%)

**Table II:** Distribution and prevalence of hypertension, obesity and overweight in blood donor

** Blood Pressure**		
**No. of Healthy Person (%)**	**No. of Hypertension Person (%)**	**Total**
18196 (78.1%)	5124 (21.9%) (p < 0.001)	23320

**Table III:** The association between hypertension, obesity and ABO blood groups

** Blood Group**				
**Parameter**	B	O	A	AB
**Hypertension** **n=5124 (21.9%)**	2018 (8.7%)	1786 (7.6%)	856 (3.7%)	464 (1.9%)
**Obesity** **n=4856 (20.9%)**	1856 (7.9%)	1428 (6.2%)	1326 (5.8%)	246 (1.0%)

**Figure 1:**
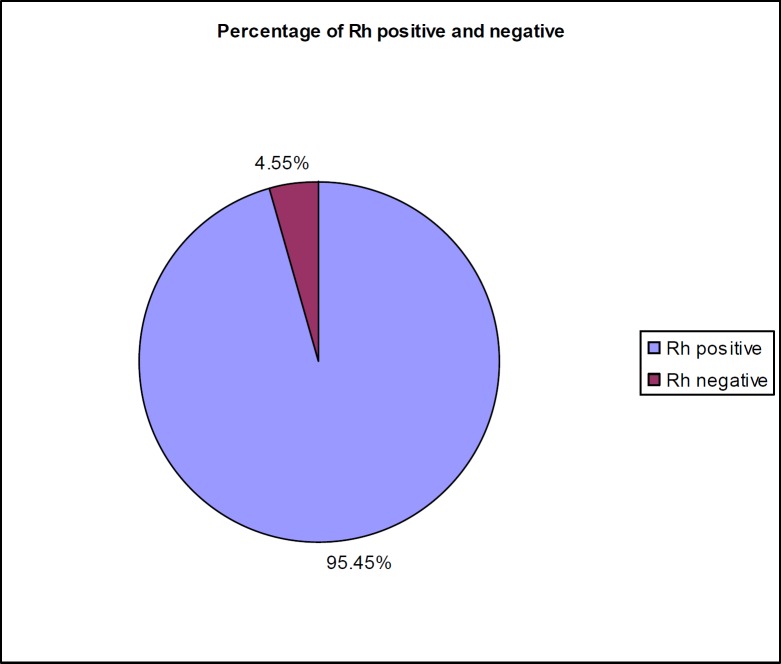
Percentage of Rh Positive and Negative blood donors

## Discussion

In the present study, the hypertension and obesity was significantly increased as compared to healthy person. We observed that the B blood group seen more in the hypertension and obesity followed by blood group O, A and AB. In our study, we found that the B blood group was more susceptible to hypertension and obesity as compared to blood group O and A; whereas AB blood group had less chance of getting hypertension and obesity. This could suggest that B group might genetically more prone to hypertension as compared to other groups. Although this is preliminary study, a clear trend is seen which is in agreement with some studies (16-18). These figures are similar to other study carried out in Iran (19). Research on ABO group system has been of immense interest, due to its medical importance in different diseases. The ABO blood group system is not only important in blood transfusions, cardiovascular diseases, organ transplantation, erythroblastosis in neonates, but also one of the strongest predictors of national suicide rate and a genetic marker of obesity (20, 21). A significant deficit of group O has suggested that there may be susceptibility to develop osteoarthrosis in normal hip-joint and spinal osteochondrosis (22, 23). The genetic history of a person can be known by studying the blood groups (24). In our study the ABO blood groups and Rh positivity in male and female donors showed that the blood group B positive was most prevalent in both male and female followed by group O, A and AB. In contrast, the blood group O is the most prevalent group in Egypt (25). Blood group A in Russian Federation (26). The commonest groups in Australians are O and A, while in Africans B group is commonest (27). In USA 46% show group O, 41% group A, 9% group B and 4% group AB (28). In Saudi Arabia, 52% are group O, 25% group A, 19% group B and 4% group AB (29). According to an Iranian study blood group O is the most common group (41.16%) over there (30).

India is a country with a lot of diversity in of race, religion and creed. Hence diversity has been observed in the distribution of blood groups in population within the country. Study from South India showed that blood group O was commonest (38.75%) followed by group B (32.69%), group A (18.85%) and AB (5.27%) (31). Similarly studies in Jammu and Kashmir also showed O to be commonest among ABO group in their population (32). These results were different from our study where B group was commonest. Our study represented mainly Uttar Pradesh populations which focus as the highest populated state of Northern India. Further we observed that none of the female donors were AB negative. In contrast a Swat (Pakistan) study showed that the blood group AB negative was 0.92% in female donors (33). This discrepancy may be due to the small number of negative donors included in our study. In Rhesus System, our study shows prevalence of Rh positive was 95.45%, while only was 4.55% was Rh negative (Figure 1). These figures are similar to other studies carried out in Maharashtra, India (34, 35). Our donor population showed Rh negativity of 4.55% as compared to 17% in Britain. This suggests that the expected frequency of Rh iso-immunization would be lower in our population than that encountered in the Britain population. Our donor population showed Rh negativity of 4.55% as compared to 17% in Britain. This suggests that the expected frequency of Rh iso-immunization would be lower in our population than that encountered in the Britain population. 

## Conclusion

To conclude, the B blood group in blood donor was more susceptible to hypertension and obesity. The commonest ABO blood group was group B in blood donors with Rh negativity at only 4.55%. This was in contrast to the prevalence of ABO and Rh blood groups in other parts of the world as well as also within the country.
